# The NF-*κ*B1 is a key regulator of acute but not chronic renal injury

**DOI:** 10.1038/cddis.2017.233

**Published:** 2017-06-15

**Authors:** Amy Fearn, Gerhard R Situmorang, Christopher Fox, Fiona Oakley, Rachel Howarth, Caroline L Wilson, Agklinta Kiosia, Michael G Robson, Derek A Mann, Anna Moles, Neil S Sheerin

**Affiliations:** 1 Institute of Cellular Medicine, Newcastle University, Newcastle Upon Tyne, UK; 2 Urology Department, Cipto Mangunkusumo National Referral Hospital/Faculty of Medicine, Universitas Indonesia, Jakarta, Indonesia; 3 MRC Transplant Centre, Kings College London, London, UK

## Abstract

The NF-*κ*B family of transcription factors is important for many cellular functions, in particular initiation and propagation of inflammatory and immune responses. However, recent data has suggested that different subunits of the NF-*κ*B family can suppress the inflammatory response. NF-*κ*B1, from the locus *nfκb1,* can inhibit transcription, acting as a brake to the recognised pro-inflammatory activity of other NF-*κ*B subunits. We tested the function of NF-*κ*B1 in an acute (nephrotoxic serum (NTS) nephritis) and a chronic (unilateral ureteric obstruction (UUO)) model of renal injury using NF-*κ*B1 (*nfκb1*^−/−^) knockout mice. Deficiency in NF-*κ*B1 increased the severity of glomerular injury in NTS-induced nephritis and was associated with greater proteinuria and persistent pro-inflammatory gene expression. Induction of disease in bone marrow chimeric mice demonstrated that the absence of NF-*κ*B1 in either bone marrow or glomerular cells increased the severity of injury. Early after UUO (day 3) there was more severe histological injury in the *nfκb1*^−/−^ mice but by day 10, disease severity was equivalent in wild type and *nfκb1*^−/−^ mice. In conclusion, NF-*κ*B1 modifies acute inflammatory renal injury but does not influence chronic fibrotic injury.

Glomerulonephritis (GN) is one of the leading causes of end stage renal disease (ESRD) accounting for up to 40% of ESRD in some countries.^1^ Many types of glomerular disease are immune-mediated, characterised by the presence of circulating autoantibodies and/or glomerular immune complex deposition^2^ (anti-glomerular basement membrane disease, membranous nephropathy, lupus nephritis, and anti-neutrophil cytoplasmic antibody-associated vasculitis) and their measurement is important for diagnosis. In addition to antibodies, lymphocytes, macrophages and fluid phase mediators of inflammation, in particular complement, have been implicated in the development of glomerular injury. The autoimmune response leads to glomerular inflammation and to a progressive decline in renal function due to glomerulosclerosis and tubulointerstitial fibrosis. Activated pro-inflammatory and pro-fibrotic mediators induce and sustain the pathophysiological events occurring within the affected kidney, including; renal vasoconstriction, immune cell infiltration, interstitial cell proliferation, accumulation of extracellular matrix proteins and eventually glomerular sclerosis and tubular atrophy. For most glomerulonephritides the trigger that initiates disease is unknown and the underlying disease mechanisms poorly understood. Current treatments for GN are nonspecific, toxic and of variable efficacy. Therefore better understanding of the biological drivers of GN is urgently needed in order to design more specific therapies.

NF-*κ*B1 (p50) is one of the five members (RelA, RelB, c-Rel, NF-*κ*B1 and NF-*κ*B2) of the NF-*κ*B transcription factor family. NF-*κ*B members can potentially generate fifteen NF-*κ*B homo- or heterodimers which will bind to *κ*B sequence elements present in the promoter or enhancer regions of target genes, regulating multiple pathways involved in cell survival and death, organ development, inflammation and immunological responses.^3^ All NF-*κ*B members share a Rel homology domain responsible for DNA-binding and dimerisation however, only three of them (RelA, RelB, c-Rel) have a C-terminal transactivation domain which is required to modulate gene transcription. NF-*κ*B1 (p50, generated from the precursor p105) and NF-*κ*B2 (p52, generated from the precursor p100) lack this domain and acquire transactivation function by either forming Rel/p50 and Rel/p52 heterodimers or associating with non-Rel coactivator proteins.^4, 5^ NF-*κ*B1 (p50) and 2 (p52) proteins can also form homodimers which have an important role as transcriptional repressors, modifying expression of NF-*κ*B target genes, including those involved in inflammation.^6, 7^

Increasing evidence suggests important roles for NF-*κ*B proteins in renal disease progression,^8^ mediating renal inflammation by promoting gene expression in different cell types, including renal cells, innate immune cells and lymphocytes.^9^ Many reports regarding NF-*κ*B function *in vivo* focus on RelA/p50 heterodimers, overlooking the complexity of this transcription factor family. Due to the potential dual role of the NF-*κ*B1 in both promoting and repressing inflammation further studies are needed in order to clarify the role of this subunit in renal disease pathophysiology.

To characterise the contribution of NF-*κ*B1 in the development of immune complex driven kidney disease we used the nephrotoxic serum (NTS) glomerulonephritis model. In this manuscript we report that animals deficient in NF-*κ*B1 (p50 and its precursor p105) had more severe glomerular injury and a persistent inflammatory response following NTS injection. A shared contribution to this phenotype by both bone marrow-derived inflammatory cells and resident renal cells was demonstrated using bone marrow chimeric mice. However, in contrast to acute inflammatory injury, absence of NF-*κ*B1 did not alter the progression of chronic injury. Therefore NF-*κ*B1 has an important role driving the early inflammatory response in infiltrating and resident renal cells during the acute phase of glomerulonephritis.

## Results

### NF-*κ*B1 deficiency exacerbates glomerular injury in NTS nephritis

**Figure 1:**
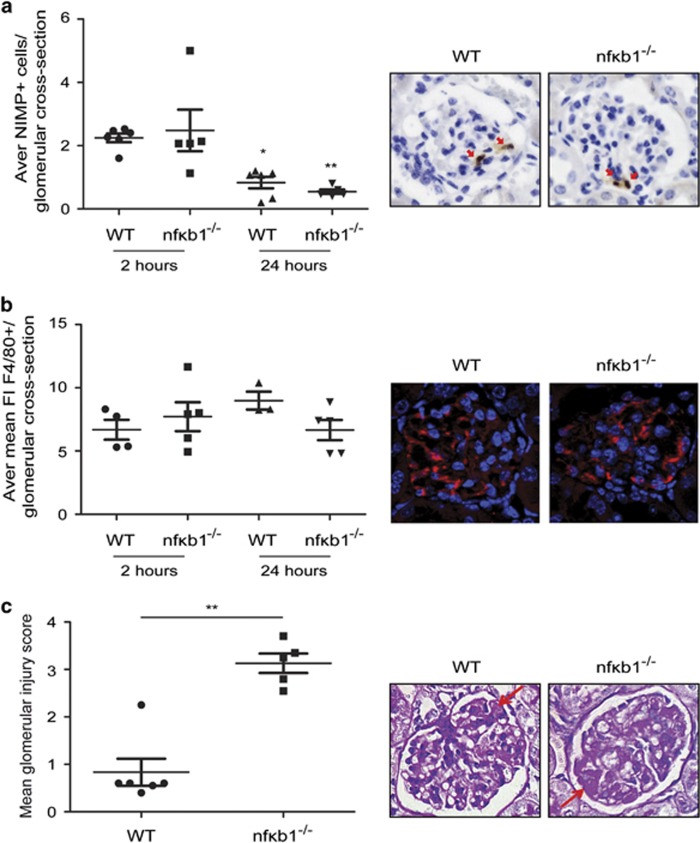
NF-*κ*B1-deficient mice have more severe glomerular injury after NTS-induced glomerulonephritis. Average number of neutrophils (NIMP+) per glomerular cross-section in WT and *nfκb1*^−/−^ renal tissues 2 and 24 h post-NTS injection and representative NIMP IHP pictures at 2 h showing neutrophil infiltration (red arrows) in a glomeruli (**a**). Average mean fluorescence intensity of F4/80+ staining per glomerular cross-section in WT and *nfκb1*^−/−^ renal tissues 2 and 24 h post-NTS injection and representative F4/80 IHP pictures at 24 h showing macrophage positive staining in a glomeruli (**b**). Glomerular injury score in WT and *nfκb1*^−/−^ mice 24 h post-NTS injection and representative PAS pictures showing areas of glomerular thrombosis (red arrows) in a glomeruli (**c**). *N*=6, unpaired *t*-test, *P⩽0.05 or **P⩽0.01

The NTS nephritis mouse model has many features which are common to clinical GN. These include, inflammatory cell influx to the kidney, proteinuria, and crescentic glomerulonephritis. In this model proteinuria and transient kidney injury is achieved by the administration of sheep anti-mouse glomerular basement membrane (GBM) heterologous antibodies which bind to target antigens in the recipient glomerulus. WT and *nfκb1*^−/−^ mice were given NTS through tail vein injection and kidneys harvested after 2 or 24 h. Neutrophil infiltration was evident 2 h after NTS injection. No differences in the number of infiltrating neutrophils (Figure 1a; Supplementary Figures 1A and B) or macrophages (Figure 1b; Supplementary Figure 1C) per glomerulus were detected between WT and n*fκb1*^−/−^ mice. Of note and consistent with previous reports in this model, 24 h after NTS injection the neutrophil infiltrate had resolved (Figure 1a).

Glomerular injury and thrombosis was evident in WT and *nfκb1*^−/−^ mice after 24 h of NTS injection. Glomerular injury was significantly worse in *nfκb1*^−/−^ mice compared with WT mice. Extensive glomerular thrombosis was only evident in *nfκb1*^−/−^ animals (Figure 1c). Therefore, lack of NF-*κ*B1 results in worse and more extensive glomerular injury during NTS-induced GN.

### Proteinuria is increased in mice deficient in NF-*κ*B1 despite equivalent binding of heterologous antibody

**Figure 2:**
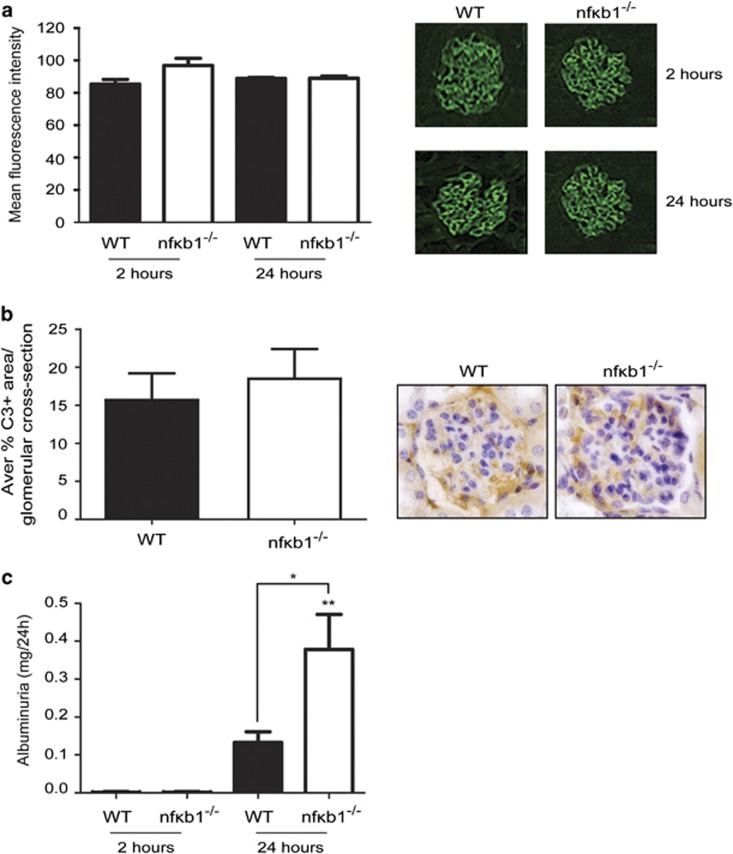
NF-*κ*B1-deficient mice have impaired glomerular function after NTS-induced glomerulonephritis despite equivalent heterologous antibody deposition. Mean fluorescence intensity quantification and representative pictures (**a**) of anti-GMB antibody binding to glomerular basal membrane of the glomeruli 2 and 24 h post-NTS injection in WT and *nfκb1*^−/−^ mice. Average of % C3+ area per glomerular cross-section in WT and nf*κ*b1^−/−^ renal tissues 2 h post-NTS injection and representative pictures showing C3 positive staining in a glomeruli (**b**). Albumin concentration in the urine collected for 24 h after 2 or 24 h NTS injection (**c**). *N*=6, one way ANOVA, *P⩽0.05 or **P⩽0.01

To confirm that the excess glomerular injury observed in the *nfκb1*^−/−^ mice (Figure 1c) was not due to a difference in nephrotoxic antibody binding, the amount of sheep IgG present in the glomeruli was quantified at 2 and 24 h post-injection. No significant differences of the mean fluorescence intensity was detected in the glomeruli of WT and *nfκb1*^−/−^ mice at 2 or 24 h indicating no differential binding of the anti-GBM heterologous antibody (Figure 2a). Complement activation, as assessed by C3 deposition in the glomeruli, was also similar in WT and *nfκb1*^−/−^ mice 2 h after NTS injection (Figure 2b).

In addition to histological glomerular injury, glomerular function was assessed by measuring 24 h urine albumin excretion. NTS injection resulted in functional injury to the glomerulus as reflected by an increase in urine albumin excretion in WT and *nfκb1*^−/−^ mice after NTS challenge (Figure 2c). The *nfκb1*^−/−^ mice exhibited significantly higher urinary albumin excretion than WT.

To determine whether *nfκb1*^−/−^ mice had an increased sustained response to injury over time, disease severity was assessed 7 days after NTS administration. Blood urea nitrogen analysis showed no differences in kidney function between WT and *nfκb1*^−/−^ mice (Supplementary Figure 2A). In addition no differences were detected in the glomerular injury score (Supplementary Figure 2B) and macrophage infiltration (Supplementary Figure 2C) in the glomeruli of WT and *nfκb1*^−/−^ mice. Taken together with the histological changes this result confirms that NF-*κ*B1 is important in preserving glomerular structure and function during acute experimental GN.

### NF-*κ*B1 deficiency increases pro-inflammatory gene expression

Disease progression on the NTS-induced nephritis model can be divided in two distinctive phases. During the first few days (or the ‘heterologous’ phase) the damage is dependent upon infiltrating neutrophils, complement activation and pro-inflammatory cytokines.^10^

**Figure 3:**
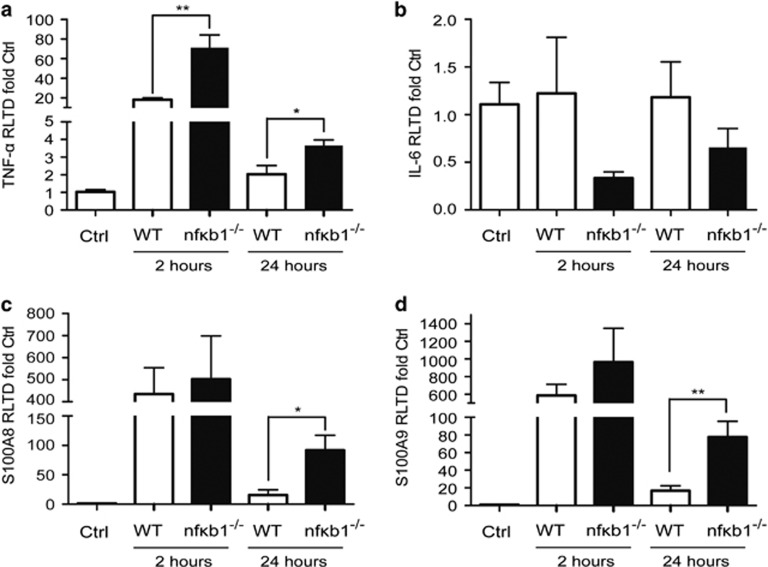
NF-*κ*B1-deficient mice have increased and sustained inflammatory responses after NTS-induced glomerulonephritis. TNF-*α* (**a**), IL-6 (**b**), S100A8 (**c**) and S100A9 (**d**) mRNA expression in kidney from saline and NTS-challenged WT and *nfκb1*^−/−^ mice at 2 and 24 h. *N*=6, repeated series of *t*-test, *P⩽0.05 or **P⩽0.01

To assess the inflammatory response of both WT and *nfκb1*^−/−^ mice we determined the levels of key pro-inflammatory proteins (TNF-*α*, IL-6, S100A8 and S100A9) which are known downstream targets for NF-*κ*B as well as recognised contributors to GN disease progression.^11, 12, 13^ TNF-*α*, S100A8 and S100A9 gene expression in kidney was upregulated 2 h after NTS challenge in both WT and *nfκb1*^−/−^ (Figures 3a) in comparison with WT saline injected animals. No difference was seen in IL-6 expression (Figure 3b). TNF-*α* levels were significantly higher in *nfκb1*^−/−^ *versus* WT mice 2 h after NTS injection (Figure 3a). Although the levels of TNF-*α*, S100A8 and S100A9 expression in *nfκb1*^−/−^ mice had fallen by 24 h, they were significantly higher than in the WT group (Figures 3a). These data are consistent with NF-*κ*B being involved in both onset and resolution of acute inflammation.^14^

### NF-*κ*B1 in both immune and resident renal cells is involved in the development of injury

**Figure 4:**
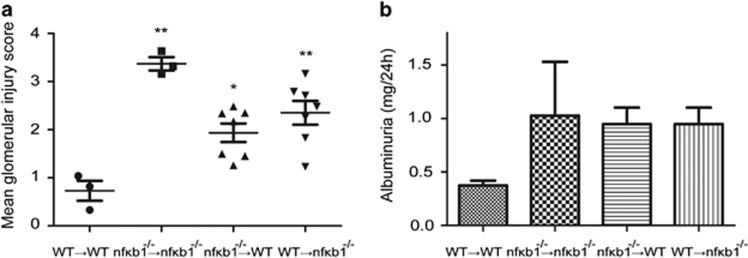
Glomerular injury is driven by the lack of NF-*κ*B1 in circulating and renal resident cells after NTS-induced glomerulonephritis. Glomerular injury score in chimeric mice WT→WT, *nfκb1*^−/−^→*nfκb1*^−/−^, *nfκb1*^−/−^→WT and WT→*nfκb1*^−/−^ (**a**). Albumin concentration in the urine collected for 24 h after 24 h NTS injection (**b**). *N*=7 for *nfκb1*^−/−^→WT and WT→*nfκb1*^−/−^, one way ANOVA, *P⩽0.05 or **P⩽0.01

NF-kB activation can have a dual role driving renal disease by regulating immune cell biological responses as well as modulating the response of resident renal cells to injury.^8^ To dissect the contribution of Nf*κ*b1 to disease development in immune and resident cells we generated bone marrow chimeric animals. Recipient mice were irradiated and transplanted with bone marrow hematopoietic progenitor cells from donor mice generating four chimeric combination: WT to WT, *nfκb1*^−/−^ to *nfκb1*^−/−^, *nfκb1*^−/−^ to WT, WT to *nfκb1*^−/−^. Peripheral blood screening confirmed bone marrow conversion to donor-type after 6 weeks (Supplementary Figures 3A and B). NTS nephritis was induced in the four chimeric groups by NTS injection. Binding of antibody to the GBM was the same across the different chimeric mice (Supplementary Figure 4). In agreement with Figures 1c and d, glomerular injury score was significantly higher in *nfκb1*^−/−^→*nfκb1*^−/−^, than in control chimeric WT→WT mice. Mice deficient in NF-*κ*B1 in either bone marrow-derived cells (*nfκb1*^−/−^→WT) or renal resident cells (WT→*nfκb1*^−/−^) developed significantly greater glomerular injury compared with control chimeric WT→WT (Figure 4a). This result suggests a role for NF-*κ*B1 in both bone marrow-derived cells and renal resident cells during the development of glomerular injury following NTS injection.

Glomerular function was assessed in chimeric mice by analysing 24 h urine albumin excretion. The level of albuminuria was higher in *nfκb1*^−/−^→*nfκb1*^−/−^ than in WT→WT (Figure 4b) consistent with worse glomerular function. Transplant of *nfκb1*^−/−^ bone marrow into WT mice (*nfκb1*^−/−^→WT) or WT bone marrow into *nfκb1*^−/−^ (WT→*nfκb1*^−/−^) led to similar levels of albuminuria as *nfκb1*^−/−^→*nfκb1*^−/−^. Therefore glomerular permselectivity is also dependent on the absence of NF-*κ*B1 in bone marrow-derived cells and renal resident cells.

### Absence of NF-*κ*B1 in either immune or resident renal cells leads to slower resolution of the acute inflammatory response to NTS-induced nephritis

**Figure 5:**
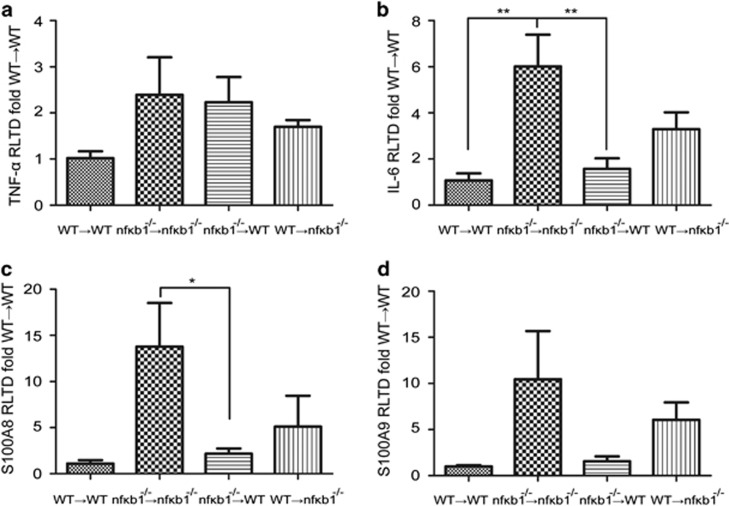
NF-*κ*B1 deficiency in both circulating and resident kidney cells regulates cytokine gene expression. TNF-*α* (**a**), IL-6 (**b**), S100A8 (**c**) and S100A9 (**d**) mRNA expression in kidney from 24 h NTS-challenged WT→WT, *nfκb1*^−/−^→*nfκb1*^−/−^, *nfκb1*^−/−^→WT and WT→*nfκb1*^−/−^ chimeric mice. *N*=7 for *nfκb1*^−/−^→WT and WT→*nfκb1*^−/−^, one way ANOVA, *P⩽0.05 or **P⩽0.01

Resolution of the inflammatory response to NTS injection was delayed in mice deficient in NF-*κ*B1 (Figures 3a–d) which could contribute to the greater glomerular injury (Figure 1c) and impaired glomerular function (Figure 2c) present in these animals. We next analysed the contribution from infiltrating inflammatory cells and resident renal cells to this delayed resolution phenotype. Gene expression levels of TNF-*α*, IL-6, S100A8 and S100A9 were analysed in the four different chimeric mice 24 h after NTS injection. As expected *nfκb1*^−/−^→*nfκb1*^−/−^ mice showed higher levels of TNF-*α*, IL-6, S100A8 and S100A9 than WT→WT (Figures 5a–d). Lack of NF-*κ*B1 in both bone marrow-derived immune cells (*nfκb1*^−/−^→WT) and kidney resident cells (WT→*nfκb1*^−/−^) was associated with higher levels of TNF-*α* in comparison to chimeric WT→WT (Figure 5a). Transplant of *nfκb1*^−/−^ bone marrow into WT (*nfκb1*^−/−^→WT) resulted in expression of IL-6, S100A8 and S100A9 (Figures 5b–d) close to WT→WT levels. WT bone marrow transplant into *nfκb1*^−/−^ showed an intermediate phenotype between *nfκb1*^−/−^→*nfκb1*^−/−^ and WT→WT. Of note, IL-6 gene expression in non-chimeric (Figure 3b) and chimeric mice (Figure 5b) after NTS challenge was different. This could be down to the presence of resident monocytic cells in the chimeric mice, which will not be affected by the chimeric switch and could influence tissue inflammation.

These results indicate that NF-*κ*B1 is responsible for gene and cell regulation and that both circulating immune cells and renal resident cells contribute to the delayed resolution response present in the *nfκb1*^−/−^ mice.

### Increased inflammatory responses in circulating and resident renal cells deficient in NF-*κ*B1

**Figure 6:**
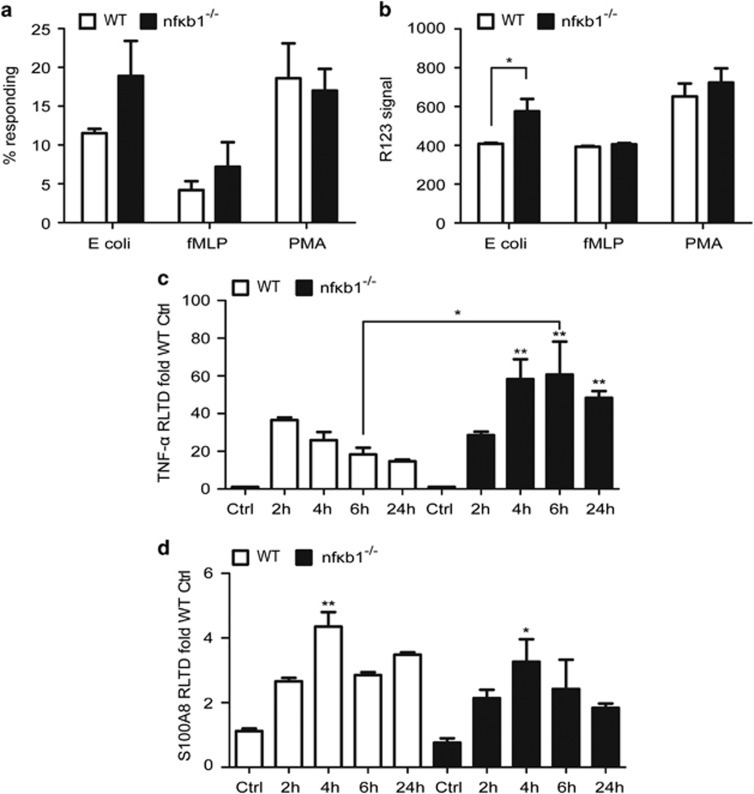
Inflammatory responses from WT and NF-kB1-deficient leukocytes and macrophages. Percentage of WT or *nfκb1*^−/−^ circulating leukocytes producing reactive oxidants (conversion of DHR123 to R123) after stimulation with *E. coli*, fMLP and PMA (**a**). Enzymatic activity per cell as expressed by R123 signal in circulating leukocytes of WT or *nfκb1*^−/−^ after stimulation with *E. coli*, fMLP and PMA (**b**). TNF-*α* (**c**) and S100A8 (**d**) mRNA expression in bone marrow-derived macrophages from WT or *nfκb1*^−/−^ treated with 100 ng/ml of LPS for 2, 4, 6 and 24 h. *N*=3, unpaired *t*-test (**a,b**), one way ANOVA (**c,d**), *P⩽0.05 or **P⩽0.01

We next explored the role of NF-*κ*B1 in circulating and resident renal cells. To assess the contribution of circulating cells we focused on bone marrow-derived macrophages and circulating leukocytes which are important effectors of the heterologous phase of NTS-induced nephritis. Circulating leukocytes from WT and *nfκb1*^−/−^ were exposed to different inflammatory stimuli. *Nfκb*1^−/−^ leukocytes had a greater oxidative response to *Escherichia coli*, but not fMLP or PMA, compared with WT (Figures 6a and b). In agreement with the gene expression levels of the NTS-challenged chimeric mice (Figures 5a–d), *nfκb1*^−/−^ bone marrow-derived macrophages stimulated with LPS showed greater and sustained levels of TNF-*α* gene expression (Figure 6c) compared with WT macrophages but similar levels of S1A008 (Figure 6d).

**Figure 7:**
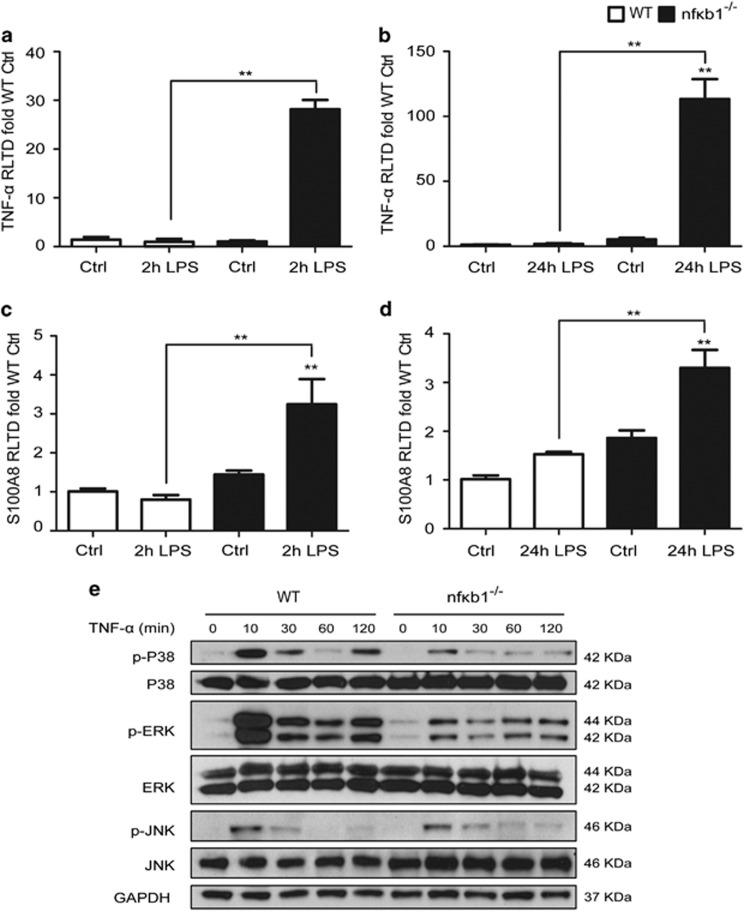
Renal resident mesanglial cells and bone marrow-derived macrophages deficient in NF-*κ*B1 present increased inflammatory responses and impaired MAPK activation respectively. TNF-*α* (**a,b**) and S100A8 (**c,d**) mRNA expression in kidney mesangial cells from WT or *nfκb1*^−/−^ after 2 h or 24 h stimulation with LPS. p-P38, P38, p-ERK, ERK, p-JNK, JNK and GAPDH protein expression in bone marrow-derived macrophages from WT or *nfκb1*^−/−^ treated with 50 ng/ml of TNF-*α* for 10, 30, 60 and 120 min (**e**). *N*=3, one way ANOVA, *P⩽0.05 or **P⩽0.01

Mesangial cells have an important role in glomerular inflammation.^15^ Mesangial cells were isolated from WT and *nfκb1*^−/−^ mice, treated with LPS for 2 and 24 h and gene expression for TNF-*α* and S100A8 was evaluated. While WT mesangial cells were almost unresponsive to LPS, *nfκb1*^−/−^ mesangial cells demonstrated a significant response at 2 and 24 h (Figures 7a–d). Our results confirm circulating and renal mesangial cells deficient in NF-*κ*B1 generate a greater inflammatory response than WT cells, pointing towards an anti-inflammatory role for NF-*κ*B1.

### NF-*κ*B1 bone marrow-derived macrophages present an impaired MAPK phosphorylation response to TNF-*α*

NF-*κ*B1 has been implicated in the regulation of MAPK signalling pathway^16, 17^ which also have an important role regulating the immune response. Therefore, we next investigated the activation of MAPK in WT and *nfκb1*^−/−^ bone marrow-derived macrophages stimulated with TNF-*α*. *Nfκb1*^−/−^ bone marrow-derived macrophages showed lower levels of P38 and ERK phosphorylation after TNF-*α* treatment (Figure 7e). In agreement, JNK phosphorylation was also lower in *nfκb1*^−/−^ bone marrow-derived macrophages than in WT. Of note, a second phosphorylation wave of P38 and ERK was observed 2 h after TNF-*α* stimulation in WT cells. This was absent in *nfκb1*^−/−^ bone marrow-derived macrophages (Figure 7e). Our results demonstrate a defective MAPK phosphorylation response in *nfκb1*^−/−^ macrophages.

### NF-*κ*B1 deficiency does not increase the severity of injury after UUO

**Figure 8:**
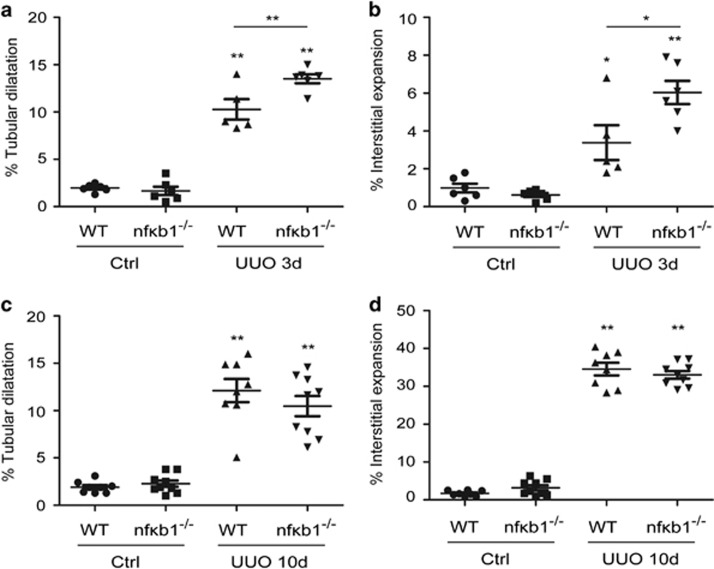
NF-*κ*B1-deficient mice show greater tubular dilation and interstitial expansion during the inflammatory phase of UUO. Percentage score of tubular dilatation and interstitial expansion in kidney tissue from WT and *nfκb1*^−/−^ controls, 3 days (**a,b**) or 10 days (**c,d**) UUO. *N*=6, one way ANOVA, *P⩽0.05 or **P⩽0.01

Our results had clearly demonstrated NF-*κ*B1 deficiency worsens acute glomerular pathology, we next assessed whether it had a similar effect on the more chronic injury associated with ureteric obstruction. To study the inflammatory and the fibrotic phase of this model we performed unilateral ureteric obstruction (UUO) in WT and *nfκb1*^−/−^ mice and harvested the kidneys at 3 and 10 days. Tubular dilatation and interstitial expansion (Figures 8a and b) was significantly higher during the inflammatory phase of the model (3 days post-UUO) in *nfκb1*^−/−^ mice *versus* WT. However, 10 days after UUO tubular dilatation and interstitial expansion were equivalent in WT and *nfκb1*^−/−^ mice (Figures 8c and d). Our results demonstrate that NF-*κ*B1 is important in the early phases of glomerular and tubular injury but deficiency does not influence the progression into the chronic/fibrotic phase.

## Discussion

The clinical manifestations of glomerulonephritis (GN) are a result of acute often persistent inflammation. Research into the immune and inflammatory basis of glomerulonephritis tend to focus on the mechanisms that drive glomerular inflammation. However, there may be pathways that regulate inflammation and therefore influence the severity of injury. In this manuscript we describe a key role for NF-*κ*B1, expressed in both resident and infiltrating cells, in the regulation of glomerular inflammation.

NF-*κ*B is a family of pleiotropic transcription factors regulating transcription of hundreds of genes related to inflammation, immunity, apoptosis, cell proliferation and differentiation. NF-*κ*B is known to be activated in both patients with kidney disease (diabetic nephropathy,^18^ glomerular disease^19^) and animal models of renal inflammation and injury.^8^ NF-*κ*B is considered to promote inflammation during renal disease with evidence correlating NF-*κ*B activation to the severity of renal disease.^18, 20^ However, most of the reports are limited to the function of the NF-*κ*B heterodimer, RelA/p50, while the function of other NF-*κ*B complexes in renal disease is still not clear. It has been suggested that the NF-*κ*B1 subunit, p50, which is encoded by *nf-κb1* gene, has a dual role in many pathological processes promoting inflammation as heterodimer (RelA/p50) or repressing it as homodimer (p50/p50).^21^

Here we investigate the role of NF-*κ*B1 in NTS-induced GN mouse model. Our results demonstrate *nfκb1*^−/−^ mice, which are deficient in p50 subunit and its precursor p105, had increased glomerular injury (Figures 1c and 2c) 24 h after NTS injection compared with WT mice. Similar levels of infiltrating neutrophils (Figure 1a; Supplementary Figures 1A,B), macrophages (Figure 1b; Supplementary Figure 1C) and C3 deposition (Figure 2b) were seen at the site of injury suggesting an equivalent immediate response to heterologous antibody, in WT and *nfκb1*^−/−^ mice but the subsequent injury that developed at 24 h was more severe. Our results are consistent with the proposed protective role of p50 in rat models of ischaemia reperfusion^22^ and anti-Thy1 glomerulonephritis.^23^ Prolonged NTS challenge (7 days) did not demonstrate delayed injury resolution in *nfκb1*^−/−^ mice (Supplementary Figures 2A–C), demonstrating an important function for NF-*κ*B1 during acute but not chronic experimental GN.

NF-*κ*B is involved in both the initiation and resolution of the acute inflammatory response^14^ having opposite roles at different stages of inflammation. During the initiation NF-*κ*B stimulates the synthesis of inflammatory mediators such as TNF-*α*, angiotensin II and monocyte chemoattractant protein-1 (MCP-1). In contrast during resolution, NF-*κ*B down-regulates inflammatory genes, up-regulates anti-inflammatory genes and induces apoptosis of leukocytes. Our assessment of inflammatory mediators in WT and *nfκb1*^−/−^ mice after 2 and 24 h of NTS challenge demonstrated an increased and sustained inflammatory response (Figures 3a–d) in *nfκb1*^−/−^ mice. The more severe phenotype observed in the *nfκb1*^−/−^ mice could be a result of the absence of p50/p50 homodimers, which are thought to act as repressors for the NF-*κ*B mediated inflammatory gene induction during experimental renal injury.^22^

During renal disease NF-*κ*B controls injury by modulating the response of infiltrating immune cells and kidney resident cells. To analyse the contribution of each of these cell types we generated bone marrow chimeras. WT mice receiving WT bone marrow had the least severe disease. The absence of NF-kB1 in either infiltrating bone marrow-derived cells or resident renal cells resulted in an increase in disease severity (Figures 4a and b). This is consistent with the hypothesis that NF-kB1 has regulatory functions in both resident and infiltrating cells.

Evidence for the regulatory function of NF-kB1 in both resident and infiltrating cells was obtained using isolated bone marrow-derived macrophages and renal mesangial cells. Macrophages from *nfκb1*^−/−^ mice had greater TNF-*α* expression compared with WT macrophages after LPS challenge (Figure 6c), whilst mesangial cells had increased expression of both TNF-*α* and S100A8 (Figures 7a–d). With the recent report of a key role for the proteins S100A8 and S100A9^13^ in glomerular inflammation and the known function of TNF-*α*, NF-kB1 may regulate multiple pathways involved in glomerular inflammation.

To have its essential role controlling inflammation, NF-kB requires the assistance of other transcription factors, such as members of the activator protein (AP)-1 family, which are dependent on mitogen activated protein kinase (MAPK) signalling pathways.^24^ Therefore we next analysed the MAPK signalling pathways in bone marrow-derived macrophages after TNF-*α* treatment. In agreement with previous reports,^16, 25^ *Nfκb1*^−/−^ bone marrow-derived macrophages showed impaired MAPK phosphorylation in comparison with WT (Figure 7e). NF-kB1 can regulate TPL-2 activity, which is the upstream kinase for the activation of the MAPKs.^26^ Further investigation will need to be done to fully understand the relative contribution of altered NF-kB function and the impaired activation of the MAPKs to the altered immune responses present in the *nfκb1*^−/−^ mice.

In addition to the phenotype in bone marrow-derived macrophages and renal mesangial cells, isolated leukocytes from *nfκb1*^−/−^ mice showed greater oxidative response than WT (Figures 6a and b), therefore infiltration of *nfκb1*^−/−^ leukocytes in the site of injury have the potential to cause greater damage. As reported previously our data suggests that NF-kB1 controls cell responses in multiple cell types.

Previous reports have also described not only a cell dependent but also a time-dependent role for NF-kB1 during acute renal injury. Absence of NF-kB1 during the first 24 h of sepsis-induced acute kidney injury reduced renal inflammation,^27^ however the lack of NF-kB1 at 48 h resulted in higher mortality and prolonged renal inflammation.^23^ To analyse the role of p50 in a more chronic injury we used the tubular injury model of unilateral ureteric obstruction or UUO. NF-kB1 absence led to increased tubular dilatation and interstitial expansion after UUO during the inflammatory phase of the injury, 3 days (Figures 8a and b). However, no differences where observed in the chronic/fibrotic phase, 10 days (Figures 8c and d). Our results demonstrate a critical protective role for NF-kB1 during the onset of acute renal injury but not the chronic phase.

The pro-inflammatory activity of NF-*κ*B has made it an attractive option for drug targeting. Therapeutic and pre-clinic efficacy has been shown in models of rheumatoid arthritis and inflammatory bowel disease.^28^ However, according to our results, future therapeutic approaches targeting NF-*κ*B will need to carefully consider the protective functions of the NF-*κ*B subunit p50 (NF-kB1) during the onset of acute renal injury.

## Materials and methods

### Animal models of renal disease

All animals were used in accordance with UK Home Office regulations and the Animals (Scientific Procedures) Act 1986. Nfkb1^−/−^ mice^29^ were bred as homozygous lines and compared with C57BL/6 WT mice. NTS nephritis was induced in WT, *nfκb1*^−/−^ or chimeric mice by tail vein injection of 200 *μ*l of NTS containing 0.5 ng/*μ*l LPS.^30^ Controls were tail vein-injected with equivalent volume of saline. Kidneys were harvested 2 h, 24 h or 7 days after injection. Mice were housed in metabolic cages for 24 h pre and post-NTS injection. Unilateral ureteric obstruction (UUO) was performed in WT and *nfκb1*^−/−^ mice as described previously.^31, 32^ Kidneys were harvested at 3 and 10 days post-UUO. A minimum of 6 animals were used in each experimental group.

### Generation of bone marrow chimeras

Bone marrow cells were harvested from the femurs of donor mice using standard techniques. Recipient mice were irradiated (10 Gy) and after 4 h injected with 10^7^ donor bone marrow cells. After 6 weeks conversion was assessed by PCR on genomic DNA isolated from blood prior to induction of NTS nephritis for 24 h. After mice were killed bone marrow was harvested to assess the presence of NF-*κ*B p50 protein in cell lysates by western blot (Supplementary Figure 3A and B).

### Glomerular injury score

Two micron sections of formalin fixed, wax embedded kidneys were stained with Periodic Acid Schiff (PAS) following standard procedure. Infiltrating neutrophils in the glomeruli were identified by their typical nuclear morphology.^33^ Glomerular thrombosis was blindly scored as ‘0’ (no injury), ‘1’ (<25% injury), ‘2’ (25–50% injury), ‘3’ (50–75% injury) and ‘4’ (75–100% injury).^34^ At least 15 glomeruli (2 h NTS) or 30 glomeruli (24 h NTS) were counted per section.

### Biochemical analysis

Blood urea nitrogen (BUN) was performed in the Clinical Biochemistry department, Newcastle upon Tyne Hospitals NHS Foundation Trust.

### Measurement of urinary albumin concentration

Urine albumin concentration was measured by radial immunodiffusion in 1.2% agarose gels containing 150 *μ*l of rabbit anti-mouse albumin antibody (Abcam, Cambridge, UK) per 10 ml of gel. The diameter of precipitation rings was measured using UTHSCSA Image Tool 3.0 computer software (University of Texas Health Science Centre, San Antonio) and urinary albumin concentrations calculated from known standards.

### Interstitial expansion and tubular dilation analysis

For the UUO kidneys analysis of interstitial expansion and tubular dilatation was performed by superimposing a 10 × 10 grid over each of 20 non-overlapping 250 × cortical images for each section. Intersections overlaying either interstitial space or tubular luminae were counted and expressed as a percentage of the total area containing 81 grid intersections.

### Immunohistochemical staining and analysis

Immunohistochemistry was performed in 4 *μ*m formalin-fixed kidney slides. Sections were deparaffinised and endogenous peroxidase was blocked with 2% hydrogen peroxide/methanol. Pronase, Proteinase K and heat mediated sodium citrate antigen retrieval was performed for NIMP, F4/80 and C3 staining respectively. Afterwards endogenous avidin and biotin were blocked. Blocking solution and primary antibody was added overnight (NIMP, F4/80 and C3 from Abcam). After the addition of secondary biotinylated antibody and ABC, sections were developed with DAB, counterstain with Mayer’s haematoxylin and mounted with Pertex. NIMP+ cells were manually counted in at least 15 random glomeruli per section. F4/80 and C3 morphometric image analysis was performed at 400 × using Nikon Eclipse Upright microscope and NIS-Elements BR Analysis software from Nikon. At least 10 random glomeruli were analysed per kidney and data is expressed as percentage of positive staining per glomeruli.

### Immunofluorescent staining

Kidney sections were deparaffinized and hydrated. Proteinase K antigen retrieval was performed. Blocking solution was added followed by F4/80 antibody (Abcam) overnight. After washing, secondary Alexa Fluor 568 was added and slides were mounted with Prolong mounting medium. Image analysis was performed with a Zeiss Axioimager Apotome microscope and Image J Software (National Institute of Health, USA). Data is expressed as average mean fluorescence intensity in the glomeruli.

### NTS glomerular binding assessment

Glomerular binding of NTS was visualised using FITC conjugated donkey anti-sheep antibody on unfixed kidney cryosections blocked with 5% horse serum. Slides were imaged using a Leica LMD microscope. Fluorescence intensity was analysed using Image J software.

### Mesangial cell isolation and culture

Mesangial cells were isolated as described before.^35^ Briefly, WT or nfkb1^−/−^ kidney cortex was mince and passed through 100 *μ*m followed by 40 *μ*m cell strainers. The material that would not pass through the strainer was collected, centrifuged and the pellet was digested with collagenase IV for 10 min at 37 °C. Glomeruli cell outgrowths were passaged four times before obtaining mesangial cells. Mesangial cells were culture in RPMI 1640, supplemented with 10% FBS, 100 U/ml penicillin, 100 *μ*g/ml streptomycin and 1% insulin, transferrin, selenium and maintained at 37 °C at an atmosphere of 5% CO_2_.

### Bone marrow-derived macrophage isolation and culture

Bone marrow cells were isolated from femurs of WT or nfkb1^−/−^ mice and differentiated into macrophages as described previously.^21^ Cells were serum starved before treating them with 100 ng/ml of LPS for 2, 4, 6 and 24 h or 50 ng/ml of TNF-*α* for 10, 30, 60 and 120 min.

### Measurement of oxidative burst

The oxidative burst in leukocytes was measured in heparinized whole blood from WT and nfkb1^−/−^ mice using Phagoburst kit (Glicotope Biotechnology) according to manufacturer’s instructions. Using flow cytometry this kit allows the detection of percentage of phagocytic cells which produce reactive oxidants (conversion of DHR123 to R123) and their enzymatic activity (amount of R123 per cell) against several stimuli opsonized bacteria (*E. coli*), Phorbol 12-myristate 13-acetate (PMA) and N-Formyl-Met-Leu-Phe (fMLP).

### Analysis of gene expression

Total RNA was isolated from homogenised renal cortex by standard chloroform extraction followed by isopropanol precipitation. cDNA was synthesised from 250 ng of total RNA. Real time PCR was performed with SYBR Green JumpStart ready mix according to manufacturer’s instructions. Data was calculated using ΔΔCt and GAPDH was used as a housekeeping gene. Data is plotted against WT control group. Primer sequences used are listed in the Supplementary Table 1.

### Statistical analysis

Results are expressed as mean±S.E.M. unless otherwise stated in the figure legend. All *P* values were calculated using one way ANOVA followed by Bonferroni's test or two-tailed unpaired student’s *t*-test. *P⩽0.05 or **P⩽0.01 was considered statistically significant.
