# Supplementary material for: The NF-κB1 is a key regulator of acute but not chronic renal injury
Source: Cell Death Dis. 2017 Jun 15;8(6):e2883–. doi: 10.1038/cddis.2017.233 (PMC5584573; doi:10.1038/cddis.2017.233)
Supplement: Supplementary Information [file cddis2017233x1.pdf]

The NF-κB1 is a key regulator of acute but not chronic renal injury

Fearn A[1], Situmorang GR[1,2], Fox C[1], Oakley F[1], Howarth R[1], Caroline CL[1], Kiosia A[1], Robson MG[3], Mann DA[1], Moles A[1#], Sheerin NS[1# *]

[1]Institute of Cellular Medicine, Newcastle University, UK. [2]Urology Department, Cipto Mangunkusumo National Referral Hospital/Faculty of Medicine, Universitas Indonesia, Jakarta, Indonesia.[3]MRC Transplant Centre, Kings College London, UK.

[#] Both senior authors contributed equally

*Corresponding author: Neil Sheerin, Institute of Cellular Medicine. William Leech Building. Newcastle University. Newcastle upon Tyne, UK. neil.sheerin@ncl.ac.uk

SUPPLEMENTAL FIGURE LEGENDS

Supplemental figure S1: NF-κB1 deficient mice have more severe glomerular injury after NTS-induced glomerulonephritis

Average number of neutrophils per glomerular cross-section in WT and nfκb1$^{-/-}$ renal tissues 2 hours post-NTS injection **(A)**. Representative PAS pictures showing neutrophil infiltration (black arrows) in a glomeruli **(B)**. Average of % F4/80+ area per glomerular cross-section in WT and *nfκb1*$^{-/-}$ renal tissues 2 and 24 hours post-NTS injection and representative F4/80 IHP pictures at 24 hours showing macrophage positive staining in a glomeruli **(C)**. N=6, unpaired t-test, *P ≤ 0.05 or **P ≤ 0.01.

Supplemental figure S2: NF-κB1 deficient mice have similar kidney function and glomerular injury than WT in a prolonged NTS-induced glomerulonephritis model

Blood urea nitrogen (BUN) in serum from WT and nfκb1$^{-/-}$ tissues 7 days post-NTS injection **(A)**. Glomerular injury score in WT and nfκb1$^{-/-}$ mice 24 hours post-NTS injection **(B)**. Average of % F4/80+ area per glomerular cross-section in WT and *nfκb1*$^{-/-}$ renal tissues 7 days post-NTS injection **(C)**. N=6, unpaired t-test, *P ≤ 0.05 or **P ≤ 0.01.

Supplemental figure S3: Bone marrow conversion to donor-derived cells in chimeric mice.

PCR of genomic DNA from peripheral blood **(A)** of WT → WT, *nfκb1*$^{-/-}$ → *nfκb1*$^{-/-}$, *nfκb1*$^{-/-}$ → WT and WT → *nfκb1*$^{-/-}$ mice. Western blot against NF-κB1

(p105 and p50) and loading control of bone marrow macrophage cell lysates **(B)** of WT → WT, *nfκb1*$^{-/-}$ → *nfκb1*$^{-/-}$, *nfκb1*$^{-/-}$ → WT and WT → *nfκb1*$^{-/-}$ mice.

Supplemental figure S4: Anti-GBM antibody binding to the glomeruli of four different chimeric backgrounds 24 hours after NTS-injection

Mean fluorescence intensity quantification of anti-GMB antibody binding to glomerular basal membrane of the glomeruli 24 hours post-NTS injection in WT → WT, *nfκb1*$^{-/-}$ → *nfκb1*$^{-/-}$, *nfκb1*$^{-/-}$ → WT and WT → *nfκb1*$^{-/-}$ mice.

Supplemental figure S1: NF-κB1 deficient mice have more severe glomerular injury after NTS-induced glomerulonephritis

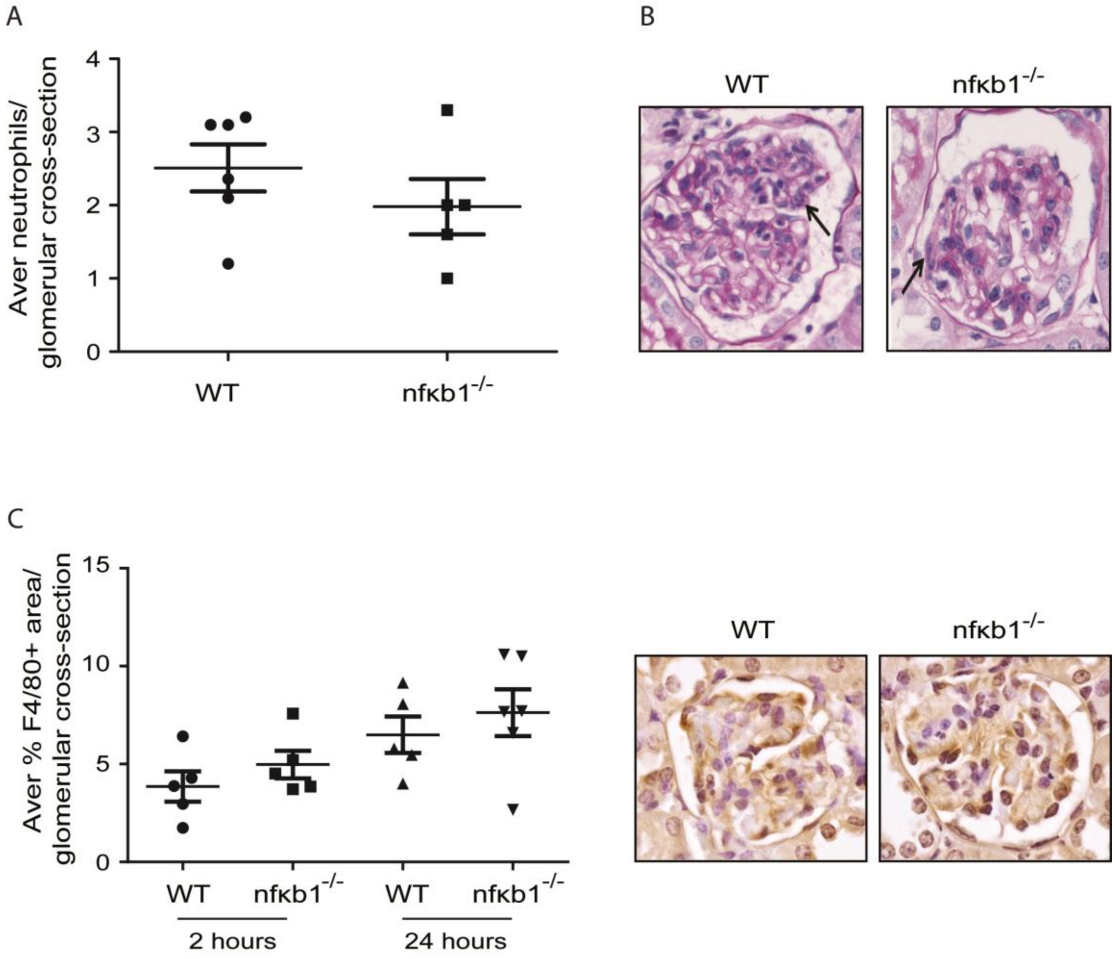

Supplemental figure S2: NF-κB1 deficient mice have similar kidney function and glomerular injury than WT in a prolonged NTS-induced glomerulonephritis model

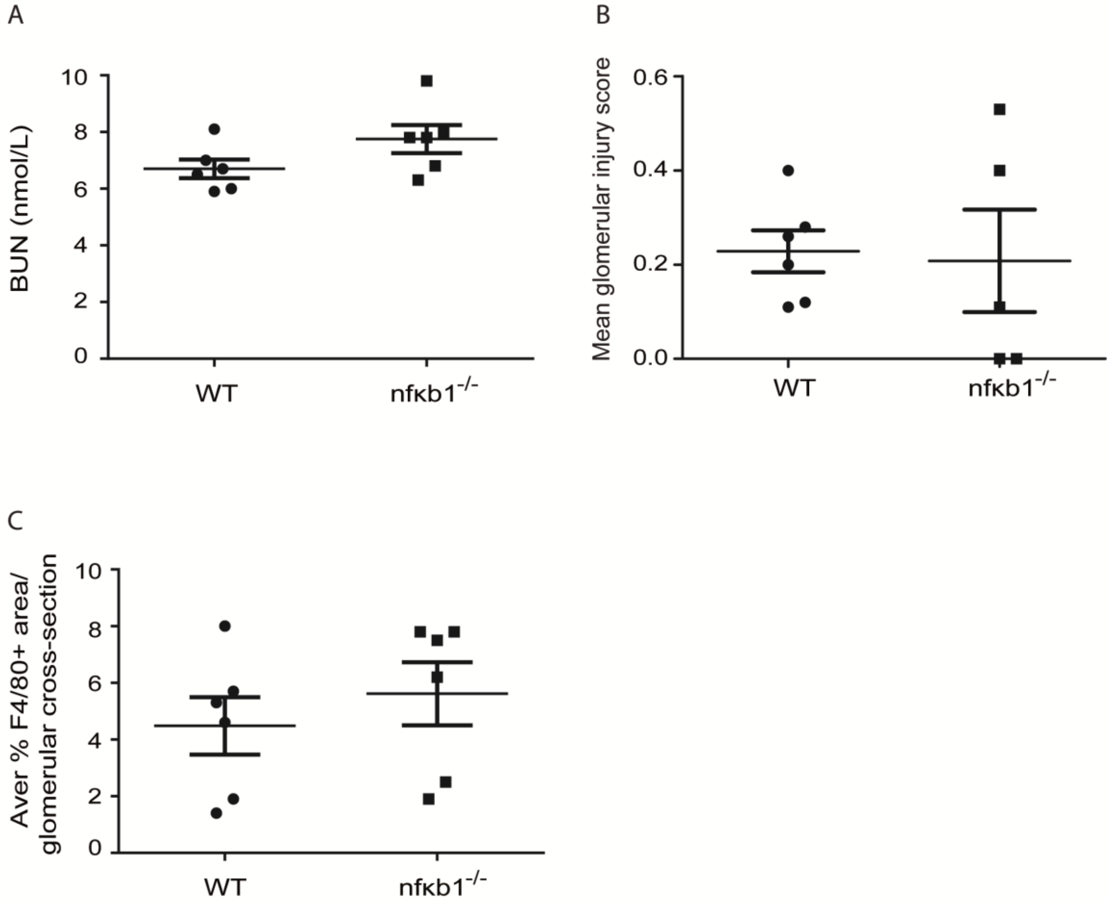

Supplemental figure S3: Bone marrow conversion to donor-derived cells in chimeric mice

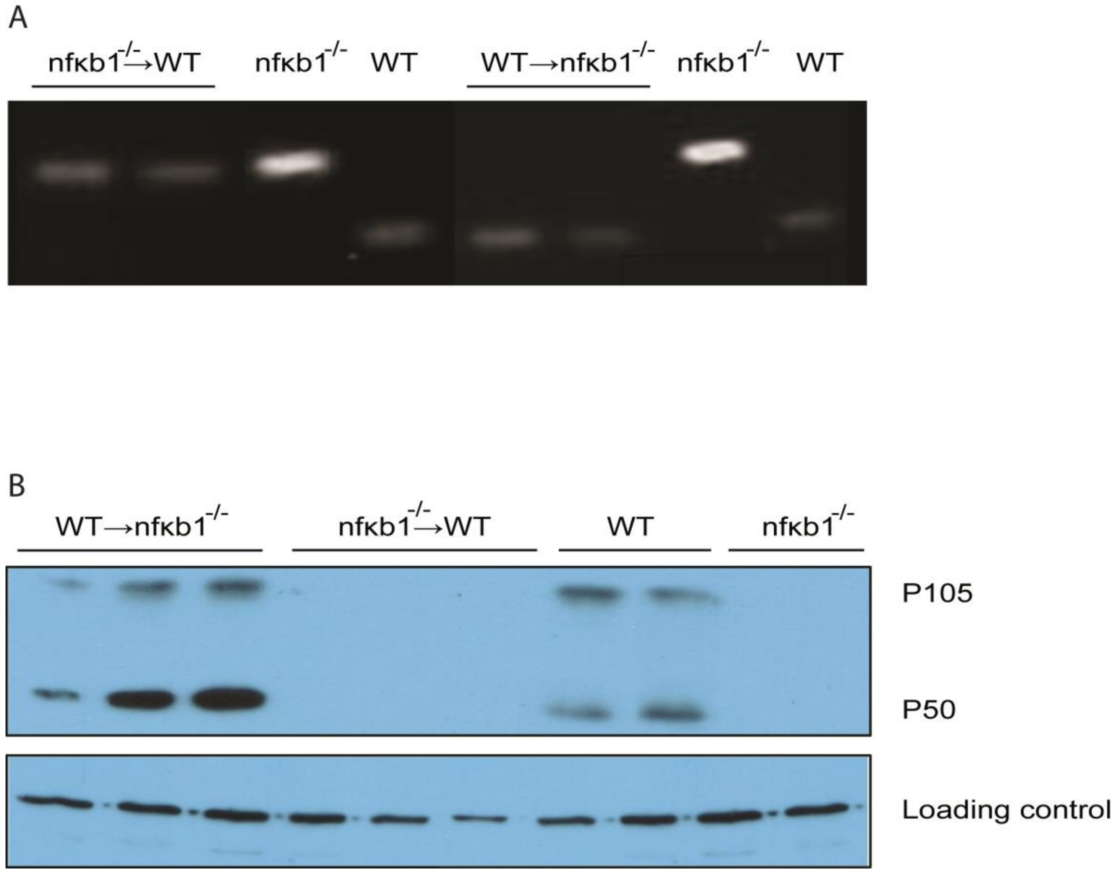

Supplemental figure S4: Anti-GBM antibody binding to the glomeruli of four different chimeric backgrounds 24 hours after NTS-injection

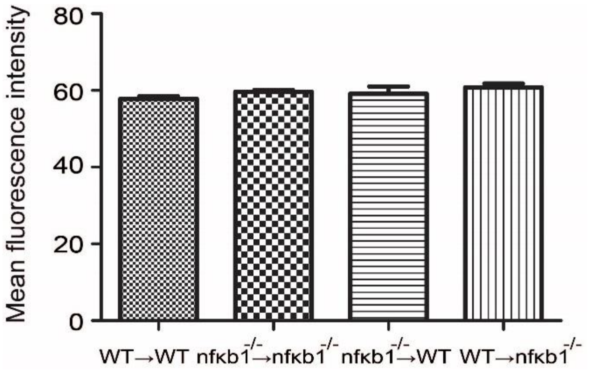

Supplementary table 1: Mouse primer sequences

Gene (GenBank Accession)	Primer sequence
GAPDH (NM_008084)	Fw: 5’-GCACAGTCAAGGCCGAGAAT-3’
	Rv: 5’- GCCTTCTCCATGGTGGTGAA-3’
IL-6 (NM_031168)	Fw: 5’- TAGTCCTTCCTACCCCAATTTCC-3’
	Rv: 5’- TTGGTCCTTAGCCACTCCTTC-3’
p50 (NM_008689)	Fw: 5’-TGGCTTTGCAAACCTGGGAA-3’
	Rv: 5’-AATACACGCCTCTGTCATCCGT -3’
S100A8 (NM_013650)	Fw: 5’- TGCGATGGTGATAAAGTGG-3’
	Rv: 5’- GGCCAGAAGCTCTGCTACTC-3’
S100A9 (NM_009114)	Fw: 5’-CACCCTGAGCAAGAAGGAAT-3’
	Rv: 5’-TGTCATTTATGAGGGCTTCATTT-3’
TNF-α (NM_013693)	Fw: 5’-CCCTCACACTCAGATCATCTTCT-3’
	Rv: 5’- GCTACGACGTGGGCTACAG-3’
